# Construction of prognostic predictor by comprehensive analyzing alternative splicing events for colon adenocarcinoma

**DOI:** 10.1186/s12957-020-02010-7

**Published:** 2020-09-03

**Authors:** Yaqi Qu, Yujia Chen, Le Zhang, Lifei Tian

**Affiliations:** 1 Department of General Surgery, Shaanxi Provincial People’s Hospital, Xi’an, 710068 China; 2 School of Basic Medical Sciences, Hebei Medical University, Shijiazhuang, Hebei China; 3 Department of Function, Shaanxi Provincial People’s Hospital, Xi’an, China

**Keywords:** Colon adenocarcinoma, Alternative splicing, Prognostic risk score, Differentially expressed genes

## Abstract

**Background:**

Colon adenocarcinoma (COAD) is one of the most common malignant tumors, with high incidence and mortality rates worldwide. Reliable prognostic biomarkers are needed to guide clinical practice.

**Methods:**

Comprehensive gene expression with alternative splicing (AS) profiles for each patient was downloaded using the SpliceSeq database from The Cancer Genome Atlas. Cox regression analysis was conducted to screen for prognostic AS events. The R package limma was used to screen differentially expressed genes (DEGs) between normal and tumor samples in the COAD cohort. A Venn plot analysis was performed between DEGs and prognostic AS events, and the DEGs that co-occurred with prognostic AS events (DEGAS) were identified. The top 30 most-connected DEGAS in protein–protein interaction analysis were identified through Cox proportional hazards regression to establish prognostic models.

**Results:**

In total, 350 patients were included in the study. A total of 22,451 AS events were detected, of which 2004 from 1439 genes were significantly associated with survival time. By overlapping these 1439 genes with 6455 DEGs, 211 DEGs with AS events were identified. After the construction of the protein–protein interaction network, the top 30 hub genes were included in a multivariate analysis. Finally, a risk score based on 12 genes associated with overall survival was established (*P* < 0.05). The area under the curve was 0.782. The risk score was an independent predictor (*P* < 0.001).

**Conclusions:**

By exploring survival-associated AS events, a powerful prognostic predictor consisting of 12 DEGAS was built. This study aims to propose a novel method to provide treatment targets for COAD and guide clinical practice in the future.

## Introduction

Colorectal cancer (CRC) is the third most common cancer and the third most common cause of tumor-related death. The incidence and mortality rate of CRC have increased dramatically over the past three decades in China because of changes in lifestyle factors [1]. CRC includes colon adenocarcinoma (COAD) and rectal adenocarcinoma. COAD is one of the main causes of cancer-associated deaths worldwide [2]. Approximately 41% of all CRCs occur in the proximal colon, with approximately 22% involving the distal colon and 28% involving the rectum. The prognosis of COAD is less than satisfactory; over 1.8 million new COAD cases and 881,000 deaths occurred in 2018 [3]. Therefore, there is a need to discover new molecules capable of identifying pre-cancerous lesions.

Alternative splicing (AS), a post-transcriptional process that modifies more than 90% of all human genes, contributes significantly to the diversity of the transcriptome and the resultant proteome [4, 5]. Exonic regions of the pre-mRNA are spliced together, and intronic regions are removed to form the final mRNA products. There is increasing evidence that the transcript variants of a gene may have opposing roles and that widespread splicing disorders are one of the molecular markers of tumorigenesis and participate in many oncogenic processes, including proliferation, invasion and metastasis, apoptosis, hypoxia, metabolism, angiogenesis, and immune escape [6, 7].

It is generally believed that dysregulation of gene expression plays an important role in the genesis and development of tumors, and studies in this area have led to improvements in diagnosis and identification of prognostic biomarkers and new therapeutic targets by analyzing the expression level of genes in various types of cancer [8, 9]. Various studies have shown that specific siRNAs, lncRNAs, and miRNAs affect the tumorigenesis and prognosis of COAD [10, 11]. Some gene modifications such as acetylation and N6-methyladenosine are also research hotspots in the field of COAD [12, 13]. However, these studies have largely ignored AS of genes. Therefore, our understanding of functionally important splicing events that contribute to tumorigenesis and the mechanisms that lead to aberrant splicing in COAD is very limited.

In this study, we aimed to explore the role of RNA AS events in the progression of COAD. To provide a deeper insight into the tumor biology, this study further explored the relationship between those molecules and the prognosis of COAD to gain a better understanding of prognostic AS events in COAD.

## Methods

### Data collection of AS events and data analysis

The Cancer Genome Atlas (TCGA), a large-scale cancer genomic project based on next-generation sequencing and microarrays to discover molecular aberrations at the DNA, RNA, protein, and epigenetic levels, started approximately 10 years ago to discover major genetic alterations in large cohorts of selected tumors, specifically those of brain, lung, and ovarian cancers. The RNA-seq data of the COAD cohort was obtained from the TCGA data portal. The SpliceSeq tool from TCGA was used to obtain the mRNA splicing pattern, known as AS, of COAD samples from the TCGA database. In SpliceSeq, a value known as Percent Spliced In ranging from 0 to 1 was calculated for each detected AS event in a gene. The R package UpSetR (version 1.3.3) was used to generate an UpSet plot for the quantitative analysis of interactive sets between seven AS types.

Data on the clinical parameters of the COAD cohort were downloaded from TCGA. To rule out the effects of surgery leading to death, patients with incomplete clinical information and a survival time of less than 30 days were excluded from the cohort. Finally, 350 patients with COAD with full clinical follow-up data were included in the study.

### Screening for survival-associated AS events in the COAD cohort

Univariate Cox analysis was conducted to determine survival-associated AS events for COAD (*P* < 0.05).

### Screening for differentially expressed genes (DEGs) in the COAD cohort

The R package limma was used to search for DEGs between normal and COAD test subjects. The cutoff criteria were an adjusted *P* value of < 0.05 and a logFC value of ≥ 1 or ≤1. A volcano map was drawn to show the criteria for selecting differential genes using the R package ggplot2. A hierarchical clustering heat map of DEG expression was visualized with the R package pheatmap.

### Overlap between survival-associated AS genes and DEGs

The overlap between survival-associated AS genes and DEGs was screened using Funrich (http://www.funrich.org/), and Venn diagrams were used to identify DEGs with survival-associated AS. Specific genes that were differentially expressed and occurred with AS events were obtained. We termed these genes as “differentially expressed genes overlapping with AS events (DEGAS)” hereafter.

### Functional enrichment of DEGs by gene ontology (GO) and Kyoto Encyclopedia of Genes and Genomes (KEGG) analyses

Metascape (http://metascape.org/gp/index.html#/main/step1), an online analysis tool suite with the function of Integrated Discovery and Annotation, mainly provides typical batch annotation and GO term enrichment analysis to highlight the most relevant GO terms associated with a given gene. GO and KEGG pathway enrichment analyses were used to identify DEGAS using Metascape. *P* < 0.05 was considered statistically significant.

### Construction of prognostic models for the COAD cohort

The top 30 most-connected DEGAS in the protein–protein interaction (PPI) network were identified using Cytoscape (version 3.6.1). Multivariate Cox regression analysis was used to assess the prognostic value of DEGAS for overall survival (OS). We constructed a prognostic model using the sum of DEGAS expression levels weighted by the regression coefficient (*β*) from the multivariate analysis:

$$ \mathrm{RiskScore}=\sum \limits_{i=1}^n{\mathit{\exp}}_i\ast {\beta}_i $$

where *n* is the total DEGAS sequence number in the risk score, *i* is the sequence number of DEGAS in risk score, exp_i_ is the expression of DEGAS, and *β*_i_ is the regression coefficient of gene *i* in the multivariate Cox regression analysis. The median score was used as a cutoff value to classify patients with COAD into low-risk and high-risk groups.

Survival analyses were assessed using the Kaplan–Meier (KM) method and the two-tailed log-rank test. The efficiency of the predictive model was further assessed using receiver operating characteristic (ROC) curves with the survivalROC package in R. The area under the curve (AUC) of the ROC curves was calculated. All analyses were performed using R software (version 3.6.1).

The risk score was combined with clinical factors using univariate and multivariate Cox regression to identify whether it could be used as an independent prognostic predictor for survival time. The results were represented as forest plots generated by the forestplot R package.

### Networks of survival-associated AS genes and splicing factors

The expression of splicing factor genes in the mRNA splicing pathway was investigated by analysis of the level 3 mRNA-seq data in TCGA. Next, the correlation between survival-associated splicing factors and DEGAS was analyzed using the Spearman test. Cytoscape software was used to construct the interaction network. *P* < 0.01 and coefficient = 0.6 were used as the inclusion criteria.

### Data analysis and generation of graphs

## Results

### Overview of AS events in COAD

**Fig. 1:**
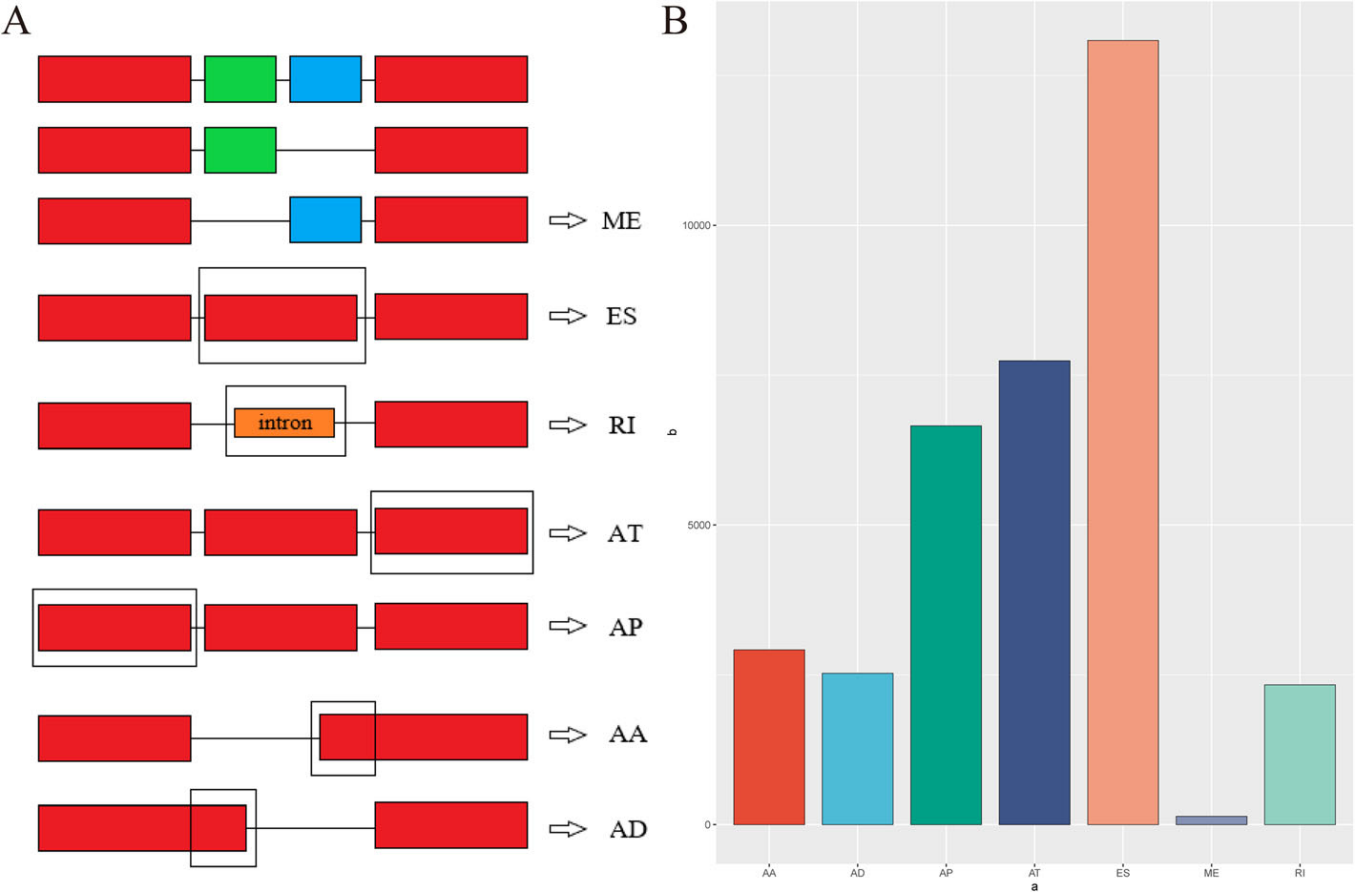
Characterization of the seven types of alternative splicing (AS) events in the study. **a** Overview of seven types of AS events: mutually exclusive exons (ME), exon skip (ES), retained intron (RI), alternate terminator (AT), alternate promoter (AP), alternate acceptor site (AA), and alternate donor site (AD). **b** Number of AS events from 350 patients with colon adenocarcinoma (COAD)

**Fig. 2:**
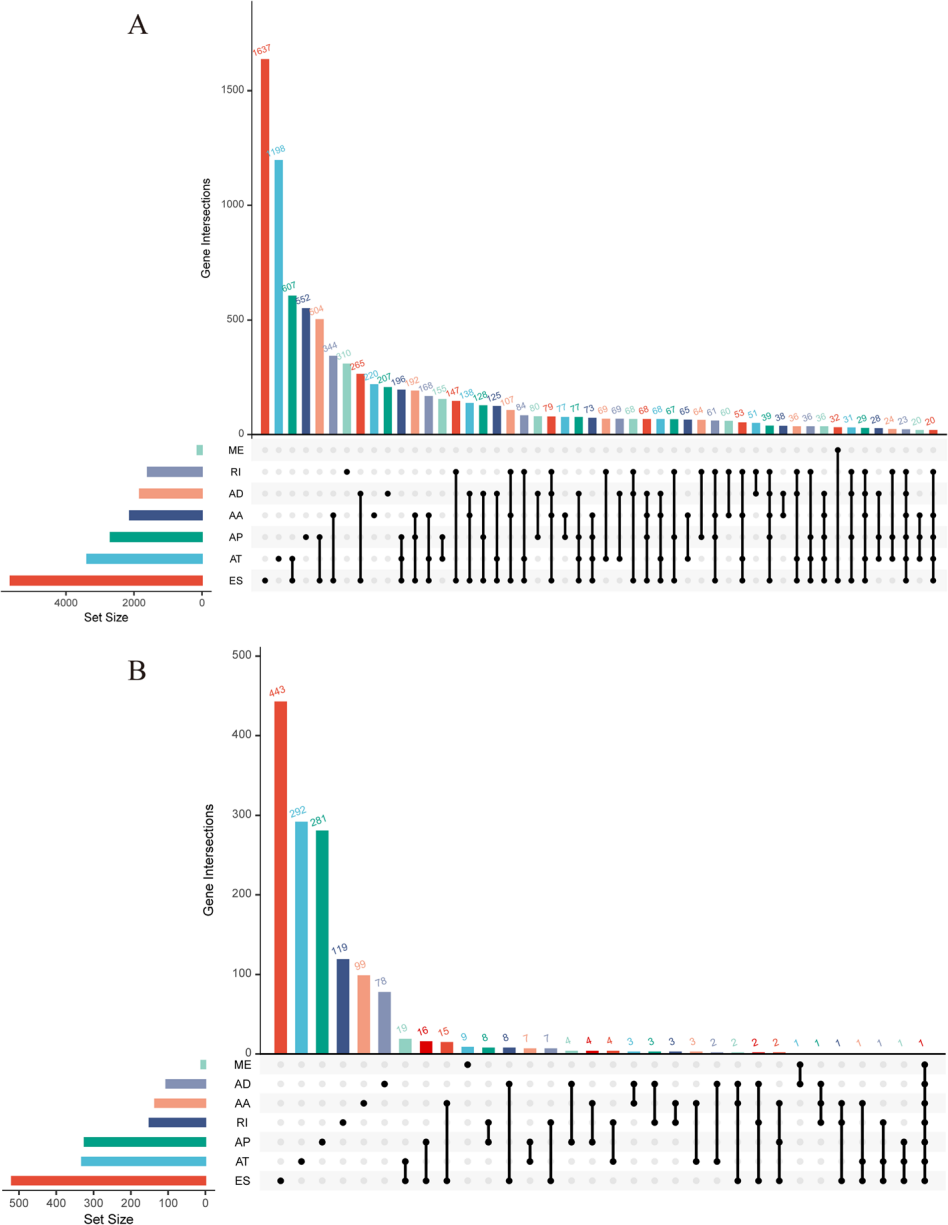
UpSet plots of alternative splicing (AS) events in colon adenocarcinoma (COAD). **a** Distributions of seven different types of AS events from 350 patients with COAD. **b** Distributions of the seven different types of survival-associated AS events in COAD

Comprehensive AS events were identified in seven splicing types: exon skip (ES), mutually exclusive exon (ME), retained intron (RI), alternate promoter (AP), alternate terminator (AT), alternate donor site (AD), and alternate acceptor site (AA) (Fig. 1a). Overall, 22,451 AS events were detected, consisting of 74 ME in 74 genes, 1661 RI in 1187 genes, 1682 AD in 1309 genes, 1999 AA in 1574 genes, 4696 AT in 2770 genes, 3972 AP in 2325 genes, and 8367 ES in 4430 genes (Figs. 1b and 2a).

### AS events associated with survival time

**Fig. 3:**
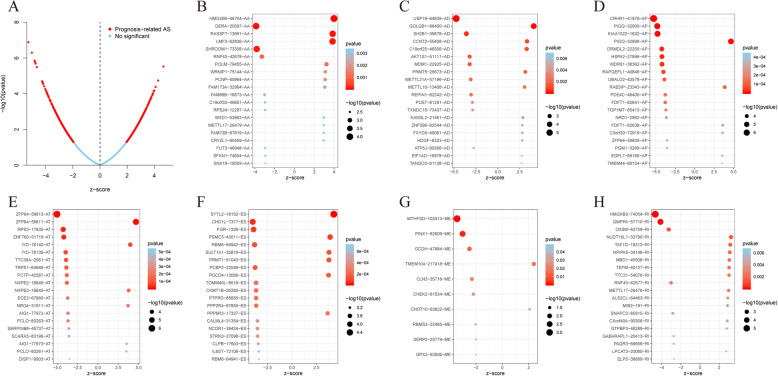
Screening for prognosis-related alternative splicing (AS) events. **a** Volcano plot showing the significance of prognosis-related AS events. **b**–**h** Bubble charts showing the significance of the top 20 prognosis-related AS events in seven different types

The results of univariate Cox regression analysis showed that 2004 AS events in 1439 genes were significantly associated with OS (*P* < 0.05), including 10 ME in 10 genes, 158 RI in 149 genes, 105 AD in 104 genes, 140 AA in 134 genes, 517 AT in 330 genes, 448 AP in 323 genes, and 586 ES in 518 genes (Fig. 2b). The top 20 of each AS type with the smallest *P* value are presented in Fig. 3. As depicted in the plot, one gene may contain more than one type of AS event.

### DEGs related to survival-associated AS events

**Fig. 4:**
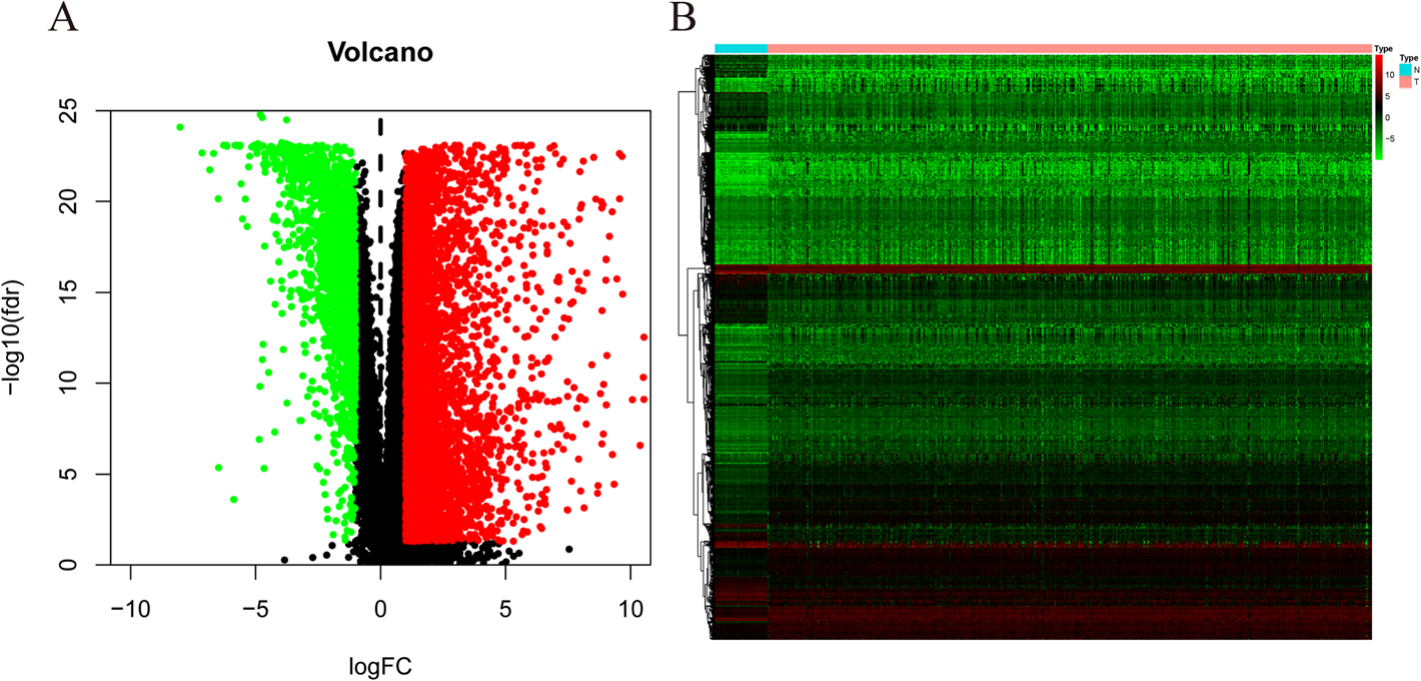
Screening for differentially expressed genes (DEGs). **a** Volcano plot showing the significance of DEGs in colon adenocarcinoma (COAD). **b** Heatmap of DEGs between tumor and normal samples

**Fig. 5:**
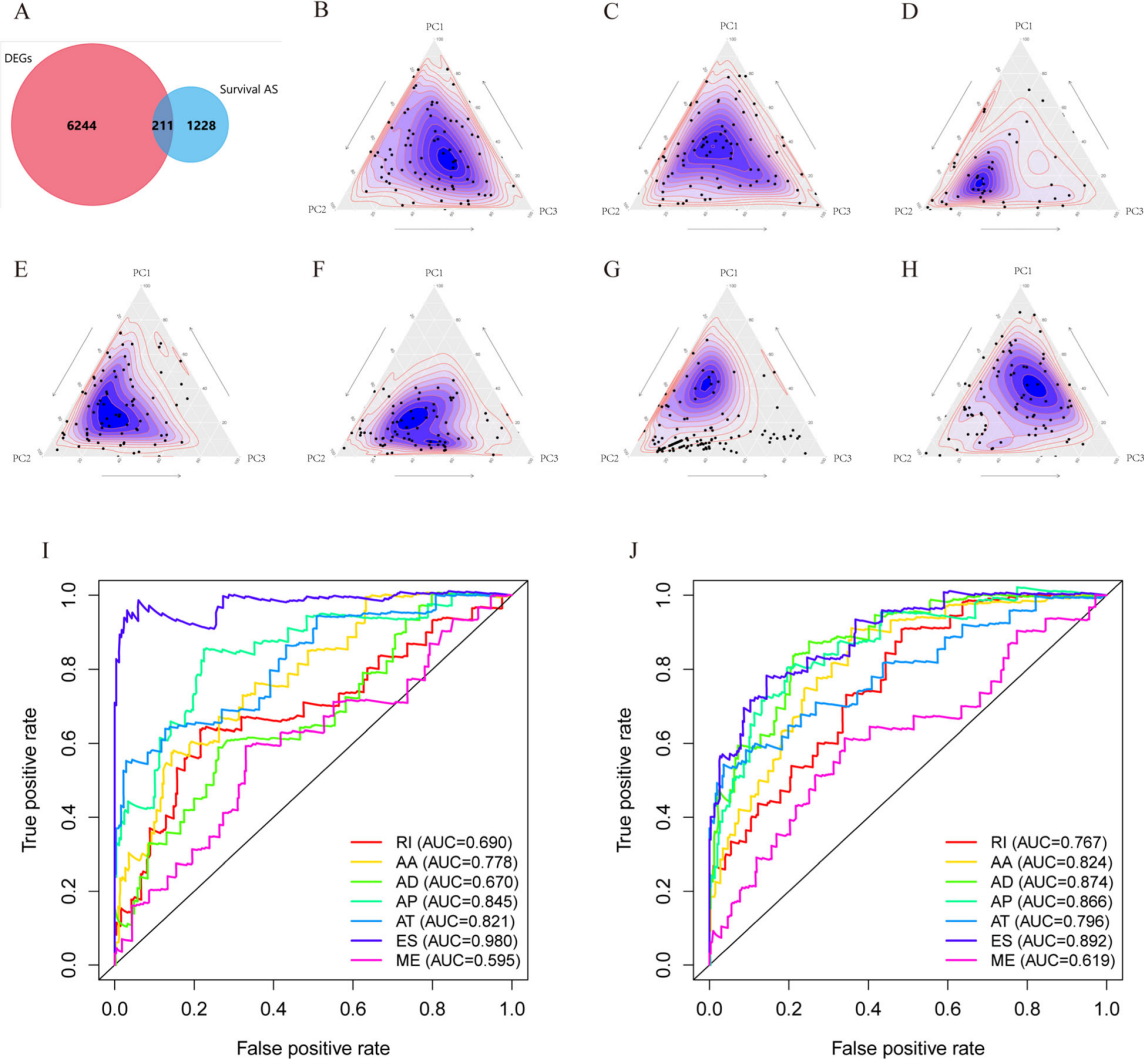
Characterization of differentially expressed genes overlapping with alternative splicing (AS) events (DEGAS). **a** Venn diagram showing the intersection of survival-associated AS events and DEGs. **b**–**h** Principal component analysis (PCA) of seven types of DEGAS. **i** Receiver operating characteristic (ROC) curves among the seven types of survival-associated AS events. **j** ROC curves of seven types of DEGAS

A total of 6455 DEGs were identified from the TCGA COAD cohort, including 4558 genes that were significantly upregulated and 1897 genes that were significantly downregulated (Fig. 4; *P* < 0.01, fold-change > 1). To identify DEGAS, we overlapped the 6445 DEGs and 1439 survival-associated AS genes. As a result, 211 DEGs were identified that overlapped with survival-associated AS events. The results are presented as a Venn diagram in Fig. 5a). Principal component analysis (PCA) was performed on the DEGAS to comprehensively characterize them (Fig. 5b–h). Multivariate Cox regression analysis was performed to evaluate the prognostic effects of DEGAS. In COAD, AD, RI, AT, AA, ES, and AP had an AUC > 0.65, and ES had the most satisfactory performance (AUC = 0.98), while ME had the worst performance (AUC = 0.595; Fig. 5i and j).

### Construction and analysis of PPI network

**Fig. 6:**
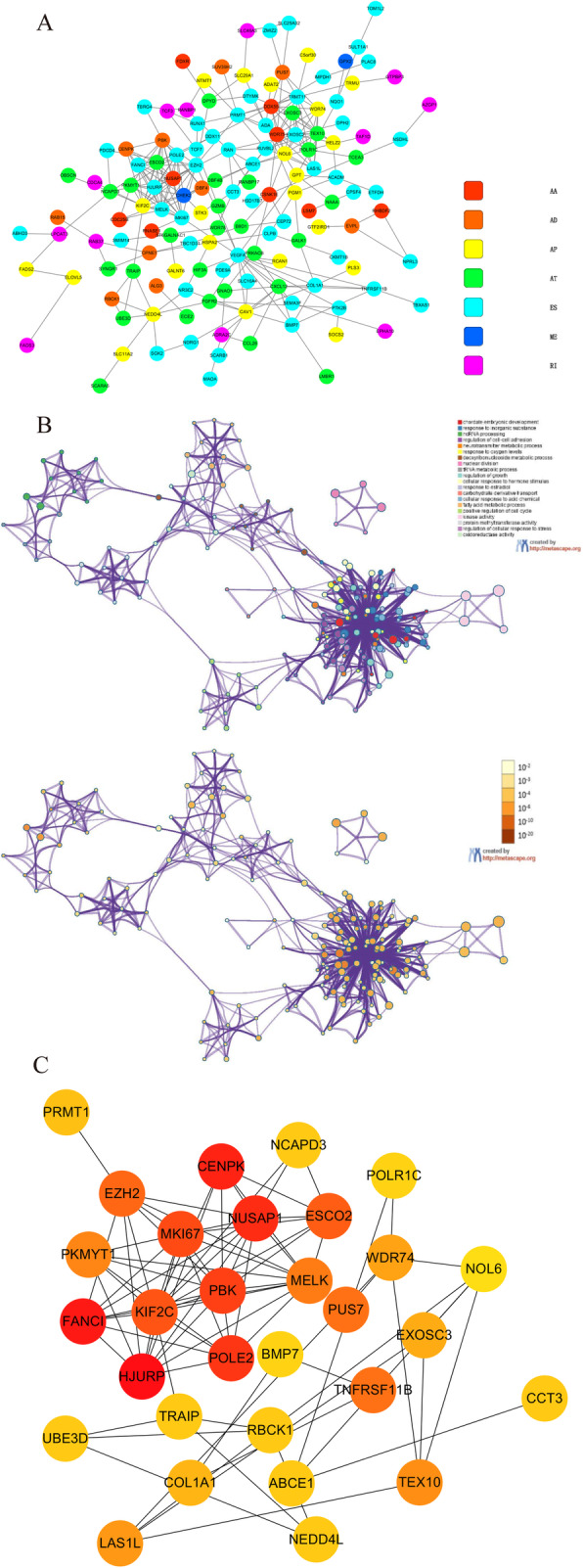
Functional enrichment analysis. **a** Gene interaction networks of differentially expressed genes overlapping with alternative splicing events (DEGAS) generated by Cytoscape. **b** Gene ontology (GO) and Kyoto Encyclopedia of Genes and Genomes (KEGG) functional enrichment analysis of DEGAS generated by Metascape. **c** Top 30 most-connected hub DEGAS in the protein–protein interaction (PPI) network

To explore the interactions among DEGAS, a string database was mapped for the most significant prognostic genes with a score > 0.4 using Cytoscape (Fig. 6a). Different types of AS events were shown in different colors. The results indicated that the majority of DEGAS showed PPIs and participated in different biological processes. The top 30 DEGAS that connected with the greatest number of nodes in the PPI network and were, therefore, considered to be the most important were identified through PPI analysis using the DMHC algorithm in Cytoscape software (Fig. 6c).

### Functional enrichment analysis

GO and KEGG analyses of the 211 DEGs were performed using Metascape (Fig. 6b). The enrichment results of GO analysis performed using Metascape showed that DEGs with survival-associated AS events were mainly enriched in chordate embryonic development, response to inorganic substances, ncRNA processing, regulation of cell–cell adhesion, neurotransmitter metabolic processes, response to oxygen levels, deoxyribonucleoside metabolic processes, nuclear division, tRNA metabolic processes, regulation of growth, cellular response to hormone stimulus, response to estradiol, carbohydrate derivative transport, cellular response to acid chemical, fatty acid metabolic processes, positive regulation of cell cycle, kinase activity, protein methyltransferase activity, regulation of cellular response to stress, and oxidoreductase activity. KEGG analysis indicated that DEGAS were mainly enriched in purine metabolism (hsa00230), fatty acid metabolism (hsa01212), cocaine addiction (hsa05030), ovarian steroidogenesis (hsa04913), longevity regulating pathway-multiple species (hsa04213), cell cycle (hsa04110), HTLV-I infection (hsa05166), RNA degradation (hsa03018), focal adhesion (hsa04510), cytokine–cytokine receptor interaction (hsa04060), and the hippo signaling pathway (hsa04390).

### Construction of prognostic models

**Fig. 7:**
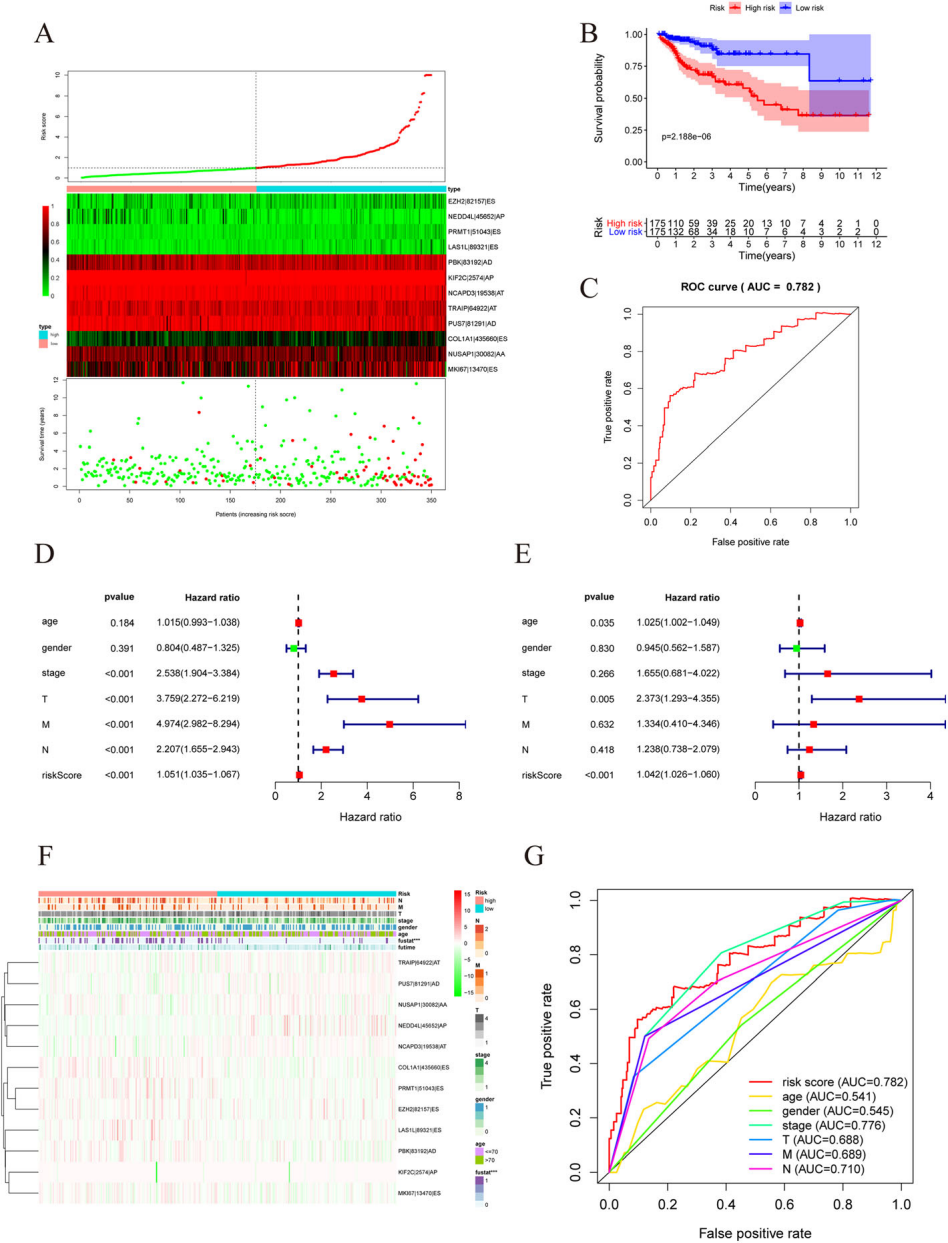
Construction of prognostic risk score model. **a** Risk score plot, heatmap of prognostic differentially expressed genes overlapping with alternative splicing events (DEGAS) in risk score, survival time, and status for patients with colon adenocarcinoma (COAD). **b** Kaplan–Meier curve of risk score. **c** Receiver operating characteristic (ROC) curve of risk score. **d** Univariate Cox analysis of clinical parameters and risk scores. **e** Multivariate Cox regression analysis of clinical parameters and risk scores. **f** Clinical parameters and risk scores of patients with COAD. **g** ROC curves with area under the curve (AUC) of clinical parameters and risk score

We obtained 30 hub DEGAS from the PPI network and used them to perform a multivariate Cox regression analysis. Regression coefficients from the multivariate Cox analysis were used to construct a risk score and develop a prognostic model to predict OS. Twelve genes (*EZH2*, coefficient = − 3.36021; *NEDD4L*, coefficient = − 3.59895; *PRMT1*, coefficient = 12.4729; *LAS1L*, coefficient = 5.054636; *PBK*, coefficient = 6.15113; *KIF2C*, coefficient = − 16.7546; *NCAPD3*, coefficient = − 9.33565; *TRAIP*, coefficient = − 3.85844; *PUS7*, coefficient = − 3.51065; *COL1A1*, coefficient = 4.254234; *NUSAP1*, coefficient = − 3.00095; *MKI67*, coefficient = 1.669178) were included in the signature. General information on the 12 genes is shown in Table 1. The risk score was defined as the sum of the expression of each gene multiplied by its coefficients. The risk score was calculated for each patient. We used the median risk score as the cutoff value to classify patients into low-risk and high-risk groups. The gene expression and survival status of each patient were ranked by the risk score values of the prognostic signature (Fig. 7a). A KM curve was plotted to indicate the correlation between the risk score and OS of patients with COAD (P_log-rank_ = 2.188e−06, Fig. 7b). The time-dependent ROC curve showed a promising performance in terms of OS prediction (*P* < 0.05, AUC = 0.782, Fig. 7c).

**Table 1:** General information of the 12 genes in the prognostic risk score

Gene stable ID	Gene name	Gene type	Chromosome	Gene start (bp)	Gene end (bp)
ENSG00000142945	KIF2C	Protein coding	1	44739704	44767767
ENSG00000183763	TRAIP	Protein coding	3	49828595	49856584
ENSG00000126457	PRMT1	Protein coding	19	49676166	49688450
ENSG00000091127	PUS7	Protein coding	7	105456501	105522271
ENSG00000001497	LAS1L	Protein coding	X	65512582	65534806
ENSG00000137804	NUSAP1	Protein coding	15	41332841	41381050
ENSG00000069399	MKI67	Protein coding	19	44742620	44760044
ENSG00000148773	PBK	Protein coding	10	128096659	128126423
ENSG00000108821	COL1A1	Protein coding	17	50184096	50201649
ENSG00000106462	EZH2	Protein coding	7	148807374	148884344
ENSG00000049759	NEDD4L	Protein coding	18	58044226	.58401540
ENSG00000151503	NCAPD3	Protein coding	11	134150113	134225461

**Fig. 8:**
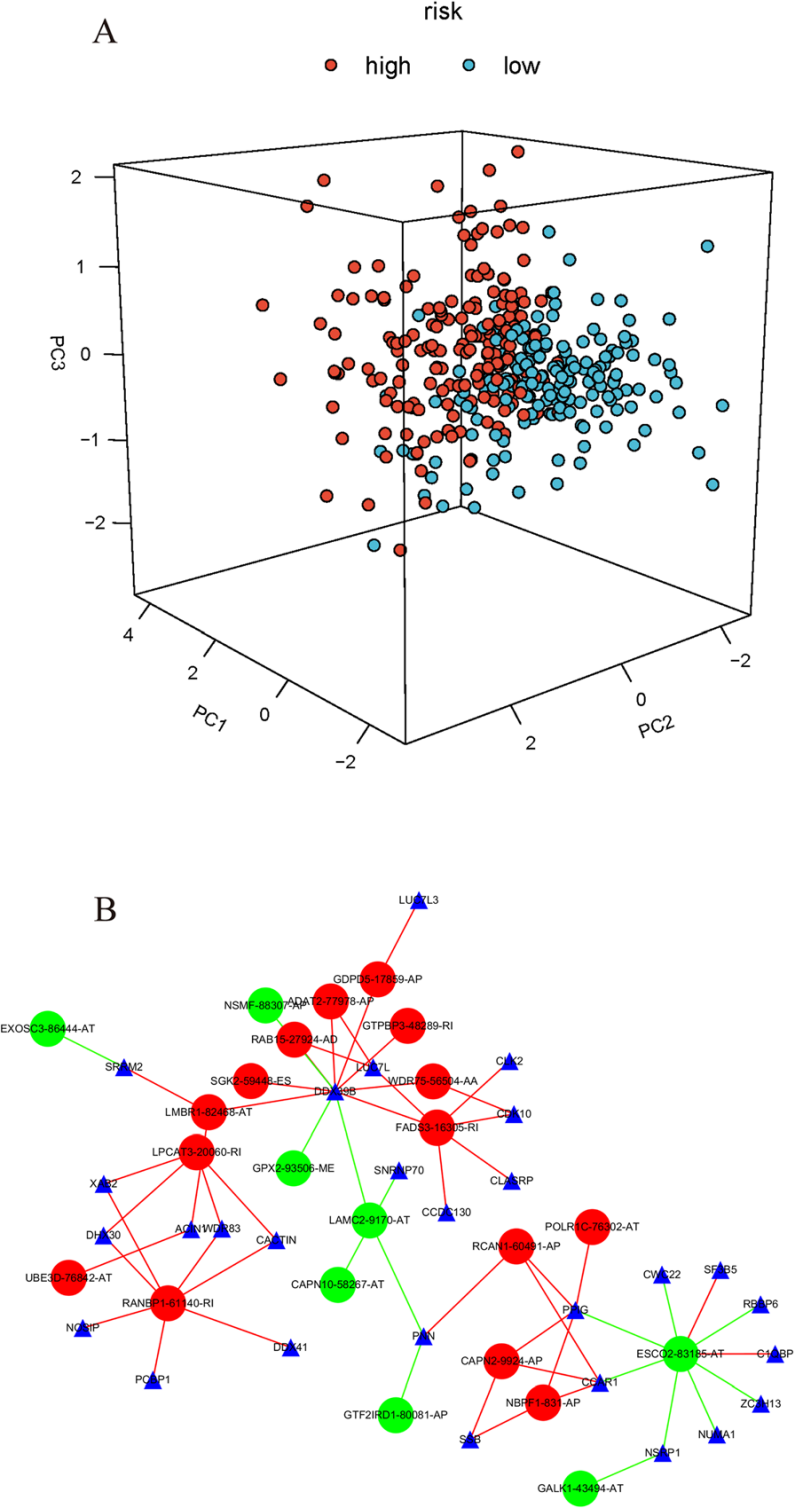
Principal component analysis (PCA) and splicing factor correlation network. **a** PCA of each patient with colon adenocarcinoma (COAD) according to risk score. **b** Splicing correlation network in COAD. Expression of 28 survival-associated splicing factors (blue triangles) was positively (red line)/negatively (green line) correlated with the Percent Spliced In (PSI) values of 12 differentially expressed genes overlapping with alternative splicing (AS) events (DEGAS) (green dots) or adverse prognosis AS events (red dots)

The results of univariate and multivariate Cox regression analysis showed that the risk score was effective at predicting the OS of patients with CRC [HR in univariate analysis = 1.051 (95% CI 1.035–1.067), HR in multivariate analysis = 1.042 (1.026–1.060); *P* < 0.001; Table 2; Fig. 7d, e]. The details of risk score together with other clinical elements based on ROC curve analysis are shown in Fig. 7g). PCA of all patients with COAD was performed to display the characteristics of each patient (Fig. 8a).

**Table 2:** Univariate and multivariate Cox regression analyses of the gene signature and overall survival of ccRCC patients

		*N*	Univariate analysis			Multivariate analysis		
			HR	95% CI	*P*	HR	95% CI	*P*
Age	≤ 60	260	1	1.236–2.322	< 0.001	1	1.008–1.906	0.045
	> 60	247	1.694			1.384		
Gender	Female	174	1	0.694–1.318	0.787	1	0.799–1.532	0.543
	Male	333	0.957			1.106		
Stage	I/II	307	1	1.495–1.792	< 0.001	1	2.510–4.941	< 0.001
	III/IV	200	1.637			3.522		
Risk score	Low	254	1	1.060–1.118	< 0.001	1	2.017–4.202	< 0.001
	high	253	1.089			2.911

### Splicing factor-regulated network construction

By analyzing level 3 mRNA-seq data from TCGA, a total of 28 splicing factors associated with the survival time of patients were identified (Fig. 8b).

## Discussion

AS is a biological process that produces many different splicing isoforms from one pre-mRNA to exert various biological functions. Abnormal AS has been identified as a molecular trigger for cancer [14]. Various studies have shown that the transcript variants of a gene may have opposing roles, and AS is known to be a key factor in cancer progression, including proliferation, apoptosis, invasion, angiogenesis, and metabolism [15, 16]. For example, an EGFR splice variant, lacks exon 4 (de4 EGFR), was found to constitutively activate downstream pathways to promote cancer cell proliferation and metastasis in glioma, prostate cancer, and ovarian cancer tissues [17]. PKM2, an embryonic splice isoform resulting from mutually exclusive AS of the PKM pre-mRNA, is re-expressed in many types of tumors and regulates cancer cell metabolism and tumor growth [18]. Because of the potential significance of AS in cancer biology, its clinical relevance in malignancies has received extensive attention. Moreover, it has been clarified recently that cancer-specific splice variants may potentially be used as diagnostic, prognostic, and predictive biomarkers, as well as therapeutic targets [19]. However, previous studies have mainly focused on single genes, and there has been no comprehensive assessment of the prognostic power of AS events in COAD. In the current study, AS profiles were analyzed and used to construct a regulatory network for COAD using TCGA data. Several prognostic AS events/genes were identified, which could provide potential treatment targets for patients with COAD.

We obtained gene expression and AS profile from TCGA database which has generated large amounts of genomics and proteomics data characterizing the molecular landscape of more than 11,000 tumors across 33 cancer types. TCGA is a multi-institutional comprehensive collection of various molecularly characterized tumor types, comprises multiple levels of tumor data, including genomic, transcriptomic, proteomic, and epigenetic data [20]. TCGA data provide unprecedented opportunities for systematic investigation of cancer mechanisms at multiple molecular and regulatory layers and has been used in numerous cancer studies [21]. Then, we investigated DEGs between normal and tumor samples. It has been shown that DEGs are related to the activation of various typical tumorigenesis-related pathways and are widely used in oncology research [22]. AS gives rise to multiple mRNA variants from a single gene, changing the expression level of the corresponding gene [23]. Therefore, we expect AS events occurring in DEGs to be particularly important in the progression of tumorigenesis, and they could, therefore, provide potential treatment targets for patients with COAD. Differential expression of mRNA amplifies or reduces the effect of AS events; therefore, we chose DEGAS as an object for research. We overlapped DEGs and survival-related AS genes to obtain DEGAS. Univariate and multivariate regression analyses were performed, and 12 DEGAS were identified. Based on 12 hub DEGAS, a prognostic risk score model was constructed. The prognostic risk score based on AS events showed a good efficacy in predicting the prognosis of patients with COAD.

Among the 12 DEGAS, *EZH2* was demonstrated to be a master regulator of cancer-associated epigenetic disorders by Makoto et al. [24]. Manipulation of *EZH2/PRC2* may, therefore, be a promising avenue for cancer therapy. Wang et al. [25] reported that *NEDD4L* plays a critical role in the regulation of cellular processes such as apoptosis, transport, and metastasis and is downregulated in several types of cancers. Song et al. [26] demonstrated that *PRMT1* expression promoted the growth of pancreatic cancer-derived cells both in vitro and in vivo and is significantly correlated with pancreatic ductal adenocarcinoma tumor size and prognosis in postoperative patients. Ma et al. demonstrated that over-expression of *PBK* promotes autophagy and enhances cisplatin resistance via the ERK/mTOR signaling pathway in high-grade serous ovarian carcinoma. Gan et al. [27] reported that *KIF2C* is a target gene of miR-325-3p, which has been shown to be a tumor suppressor in non-small-cell lung cancer. Yuan et al. [28] reported that over-expression of *NCAPD2/3* promotes the release of pro-inflammatory cytokines by modulating the IKK/NF-κB signaling pathway. Han et al. [29] reported that *TRAIP* is crucial in early mitotic progression and chromosome alignment defects in the metaphase. According to Brouwer et al. [30], *PUS7* is the most abundant post-transcriptional modification in RNA, which is primarily thought to stabilize secondary structures of RNA, and *PUS7* variants can lead to neurological defects. Liu et al. [31] showed that increased *COL1A1* expression is associated with poor survival, and *COL1A1* knockdown inhibited metastasis of breast cancer cells. Cordon et al. demonstrated that *NUSAP1* promotes invasion and metastasis of prostate cancer [32]. *MKI67*, encoding the proliferation marker protein Ki-67, is correlated with the progression of several cancers such as laryngeal squamous cell cancer, gastroenteropancreatic neuroendocrine tumors, and cervical cancer [33, 34]. These results demonstrate that most of the genes identified play an important role in the development of cancer.

In this study, we have investigated novel clinical biomarkers and therapeutic approaches, which could eventually be used to implement novel therapeutic strategies against COAD. For example, we can adjust the treatment plan by detecting the expression level of a certain molecular biomarker in the tumor site, and we can inhibit the further development of the tumor by knocking down or raising the expression level of a certain molecular biomarker. And the potential involvement of splicing factors in COAD was studied in our analysis. The splicing correlation network revealed obvious trends whereby the majority of favorable prognosis AS events were positively correlated with the expression of splicing factors, whereas adverse prognosis AS events were negatively correlated with the expression of splicing factors. Accordingly, the question of whether downregulation of specific splicing factors would give rise to reduced favorable prognosis AS events and increased adverse prognosis AS events requires further investigation through functional experiments.

## Conclusions

By analyzing the relationship between AS events/genes and patient prognosis, we found that AS events may be predictors of COAD prognosis. Twelve potential genes (*EZH2*, *NEDD4L*, *PRMT1*, *LAS1L*, *PBK*, *KIF2C*, *NCAPD3*, *TRAIP*, *PUS7*, *COL1A1*, *NUSAP1*, and *MKI67*) were identified from the interaction network and correlation analysis between AS events and gene expression. Many of these genes are involved in the development of cancer. Based on multivariate analysis for survival, the 12 genes may be used as biomarkers for the classification of COAD prognosis at AS event and gene expression levels.

## Data Availability

RNA-seq data was downloaded from the TCGA COAD cohort, and mRNA splicing pattern data was obtained by the SpliceSeq tool from TCGA. All the data was available from TCGA (https://tcga-data.nci.nih.gov/tcga/), and we are willing to provide our analysis data.

## Abbreviations

AA: Alternate acceptor site
AD: Alternate donor site
AP: Alternate promoter
AS: Alternative splicing
AT: Alternate terminator
AUC: Area under the curve
COAD: Colon adenocarcinoma
CRC: Colorectal cancer
DEG: Differentially expressed gene
DEGAS: Differentially expressed gene overlapping with alternative splicing event
ES: Exon skip
GO: Gene ontology
KEGG: Kyoto Encyclopedia of Genes and Genomes
ME: Mutually exclusive exon
PPI: Protein–protein interaction
RI: Retained intron
ROC: Receiver operating characteristic
TCGA: The Cancer Genome Atlas
